# A cesarean section scar dehiscence during the first trimester of an intrauterine pregnancy: a rare case report and literature review

**DOI:** 10.1093/jscr/rjae422

**Published:** 2024-06-21

**Authors:** Fadi Alhalak, Sultaneh Haddad, Gabriel Nasseh, Mira Nasseh, Joud Marroush, Rami Abaza, Aya AlSafadi, Majd Jehad Dakhalalah Bani Hani, George Michael Kabbabe

**Affiliations:** University Hospital of Obstetrics and Gynecology in Damascus, G76Q+3RH, Damascus, Syrian Arab Republic; Children's Hospital Damascus, Syrian Arab Republic; Stemosis for Scientific Research, Damascus, Syrian Arab Republic; University of Aleppo Faculty of Medicine, 646G+8FG, Aleppo, Syrian Arab Republic; University of Aleppo Faculty of Medicine, 646G+8FG, Aleppo, Syrian Arab Republic; Syrian Private University, M5, Damascus, Syrian Arab republic; Damascus University Faculty of Medicine, G748+VRH, Damascus, Syrian Arab Republic; Syrian Private University, M5, Damascus, Syrian Arab republic; Yarmouk University, Shafiq Irshidat st., Irbid, Jordan; University of Aleppo Faculty of Medicine, 646G+8FG, Aleppo, Syrian Arab Republic

**Keywords:** uterine rupture, cesarean section, first trimester, dehiscence

## Abstract

Uterine rupture is specified as a complete laceration of the uterine wall, including its serosa, leading to a connection between the endometrial and peritoneal chambers. It can occur in any stage of pregnancy and is considered a severe and perhaps fatal complication. A 35-year-old woman at 9 weeks of gestation with a medical history of five prior cesarean sections presented with lower abdominal pain that had lasted for 5 hr. We detected small amounts of free fluid in the Douglas pouch using ultrasound. Subsequently, a laparotomy revealed a cesarean scar dehiscence from a non-cesarean scar pregnancy. Patients who experience a uterine rupture may have vague symptoms, severe abdominal discomfort, abnormal uterine bleeding, and severe hemorrhagic shock, depending on their gestational age. Ultrasound imaging can be used to diagnose this fatal condition in addition to laparoscopy to immediately identify and treat the issue in urgent cases.

## Introduction

Uterine rupture is defined as a complete laceration of the uterine wall, including its serosa, resulting in a communication between the peritoneal and endometrial chambers, and this can occur at any stage of pregnancy [1]. It is a serious and potentially life-threatening complication [1], carrying a high risk of morbidity and mortality for the mother and the developing fetus [2].

The incidence of rupture in women with a previous cesarean section scar is 0.3% in comparison with an unscarred uterus, which is 1 in 5700 to 1 in 20 000 [3].

In fact, uterine rupture is a rare complication that can occur in the first trimester of pregnancy (3), even when risk factors are present [1].

Patients with a prior cesarean section were more likely to develop uterine rupture when trying a vaginal birth [3]. Short interpregnancy intervals, classical uterine scars, and misoprostol treatment can also raise the chance [3].

However, uterine rupture can occur in women with scarring from myomectomy, profound corneal resection, corneal pregnancy, trauma, and previous cesarean sections [3].

When uterine rupture manifests clinically, it usually involves acute severe abdominal pain and vaginal bleeding. Hemodynamic instability accompanied by tachycardia and hypotension may also be present in the patient [3].

## Case presentation

A 35-year-old woman was admitted to the hospital after experiencing lower abdominal pain for 5 hr, with a medical history of five previous cesarean sections (CS). The last cesarean section was 2 years before the administration. There was no medical or allergic history. On physical examinations, a lower abdominal tenderness was noticed. Her vital signs were within normal ranges with a blood pressure (BP) of 110/70 mmHg and a pulse rate (PR) of 86 beats per minute indicating a stable hemodynamic condition.

An ultrasound (US) scan identified an intrauterine gestational sac with cardiac activity, a caudal-rump length (CRL) of 9 weeks, and a gestational Sac Age (GS) of 9 weeks. A small amount of free fluid was noticed in the Douglas diverticulum. Laboratory test results on admission showed a hematocrit of 33.7 g/dl, hemoglobin of 302 cells/μl, and platelets of 9.7 g/L.

**Figure 1:**
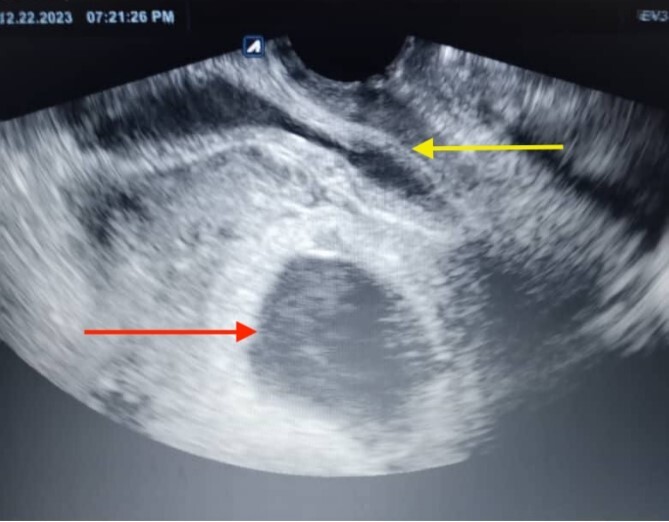
The ultrasonographic imaging conducted the day following the patient’s hospital admission revealed a ruptured gestational sac (the lower arrow), which was apparent outside the uterus. The uterine mass was visible on one side (the upper arrow), and the gestational mass was on the other side.

Furthermore, hemoglobin levels dropped to 9.7 g/L the following day and further deteriorated to 8.7 g/dl as the patient’s hemodynamic condition worsened, with her pulse accelerating to 120 beats per minute. Ultrasound revealed a moderate amount of free fluid in the pouch of Douglas and Morrison’s Pouch with an eventration in the previous cesarean scar and the gestational sac descending to the level of this scar and starting to form an occlusal line at the upper part of the uterus (Fig. 1).

**Figure 2:**
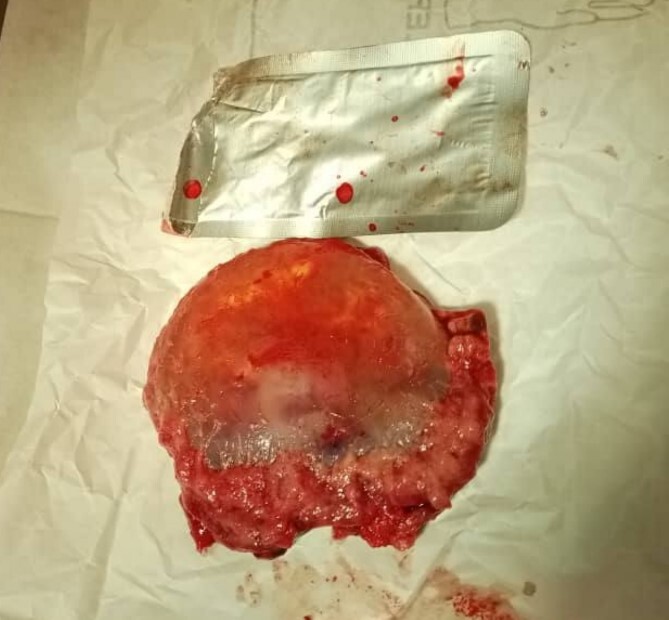
The extracted gestational sac at the end of the surgical procedure.

Given the emergent situation, a surgical procedure was initiated under general anesthesia via a Pfannenstiel skin incision. Then, it was decided to undergo a laparotomy that exposed ~1.5 L of hemorrhagic fluid and clots, followed by isolating the uterus, during which a gestational sac with the placenta, mostly protruding from the Cesarean scar, was removed along with the placental remnants (Fig. 2) (Video 1). The cesarean scar was sutured with absorbable thread, hemostasis was achieved, and the abdominal cavity was cleaned. The abdominal layers were then closed, and a drain was placed in Douglas’ pouch. During these procedures, the patient received transfusions of 3 units of complete blood and 2 units of plasma during surgery. Finally, she was discharged in good general condition 2 days later.

## Discussion

The incidence of uterine rupture among women who have had a previous cesarean section is 3 cases per 1000 deliveries. Our patient had a uterine rupture in the first trimester of her pregnancy, which is extremely rare [4]. About 1 in 500 episodes of uterine rupture end in death, making it a risky condition with a high morbidity and mortality rate [5].

Risk factors for uterine rupture include a history of uterine surgery, such as prior cesarean delivery or myomectomy. While classical incisions during cesarean deliveries are infrequent, accounting for 0.3%–0.4% of deliveries, they pose a notable risk of uterine rupture in subsequent pregnancies, with reported rates ranging from 1% to 12% based on available literature. Our patient’s medical history includes a record of five prior cesarean sections, making her susceptible to uterine rupture [6].

The induction of labor in women with previous cesarean deliveries elevates the chances of uterine rupture when compared to spontaneous labor [7]. Specifically, misoprostol induction of labor heightens the risk of uterine rupture among women who have previously undergone cesarean delivery [8]. Moreover, uterine ruptures are more common in patients having Trial of Labor After Cesarean (TOLAC) than in patients choosing scheduled repeat cesarean delivery [9]. Uterine rupture is more likely in situations where there is a low Bishop score at the time of admission to the labor and delivery unit, dystocia, delayed cervical dilatation in the first stage of labor, and an extended second stage of labor [10–12]. However, our patient did not show any signs of labor or delivery.

Limited evidence supports the association between a history of prior uterine ruptures and an elevated likelihood of subsequent occurrences; however, our patient has no history of uterine ruptures [13].

Additional risk factors include a brief inter-pregnancy interval, prior preterm cesarean delivery, and a previous cesarean delivery complicated by severe postpartum hemorrhage. Our patient’s latest cesarean delivery was 16 months before her uterine rupture [14, 15].

Patients with uterine rupture can present with a broad spectrum of symptoms and signs, ranging from nonspecific symptoms up to acute pelvic pain, metrorrhagia, and severe hemorrhagic shock, depending on their gestational age. Therefore, women in their first trimester usually present with vague symptoms [1], which can lead to a diagnostic delay. Delays in diagnosis can result in fatalities and severe bleeding. Therefore, it’s crucial to have a high index of suspicion, especially when there’s intense stomach discomfort and abnormal vital signs [3]. Particularly the same as our patient, who suffered from lower abdominal pain with a good general health condition that deteriorated several hours later.

Diagnosing uterine rupture can be done by using US imaging, which helps to detect free fluids in the peritoneum, especially in Douglas and Morrison’s pouches [3], exactly where free fluids were found in our patient.

Ultrasound might not be useful in cases of fatal bleeding; therefore, a laparoscopy or other urgent diagnostic procedures may be required to identify and treat the condition right away [3], which was done to the patient after her vital signs began to worsen.

Before now, fewer than 50 spontaneous uterine rupture cases during the first trimester and the gestational sac within the uterus have been reported in the English literature since 1990 (Table 1). With only 14 cases, the gestational age was <10, including ours. The average age was 29 years (range: 19–43 years). The most common symptom that we have detected in nearly all cases, including ours, was abdominal pain.

**Table 1:** A literature review of similar cases.

	Author	Age	Gestation age	Gravida/Para/Abortion	Symptoms	Risk factors	Rupture location	Treatment	Follow up
1	Pridjian *et al*. 1990 [16]	23	13	Primigravid	Hypotension, vaginal hemorrhage, abdominal pain	Pelvic irradiation 7 years before	Anterior fundal defect	Supracervical hysterectomy and left salpingo-oophorectomy	N/A
2	Dibbs *et al*. 1995 [17]	29	8	2/0	abdominal pain, diarrhea, and lightheadedness	placenta percreta	The fundus	Surgical repair	No complications
3	Arbab *et al*. 1996 [18]	34	13	8/1/ep5/ab1	Shock and acute abdominal pain	Placenta percreta, bilateral salpingectomy, left cornual resection	Right-sided uterine cornual rupture	Surgical repair	N/A
4	Arbab *et al*. 1996 [18]	25	8	Ab2/p2	Hemorrhagic shock	Left cornual resection, bilateral salpingectomy	The fundus	Surgical repair	N/A
5	Marcus *et al*. 1999 [19]	38	13	4/2-0-1-1	Spotting and cramping	Two cesarean scars, placenta percreta	The lower uterine segment	Radical hysterectomy	No complications
6	Hamsho *et al*. 1999 [20]	34	13	4 + 3	Abdominal pain	Four lower segment cesarean sections	The lower part of its anterior aspect	Surgical repair	No complications
7	Porcu *et al*. 2003 [21]	28	12	1/0	Acute abdominal pain	DES	Anterior fundal area	Surgical repair	No complications
8	deRoux *et al*. 1999 [22]	22	12	9/	Abdominal pain	6 curettages, placenta percreta	The fundus	Surgical repair	Died
9	Matsuo *et al*. 2004 [23]	31	10	3/1	Little abnormal genital bleeding and mild abdominal pain	Previous cesarean section, dilatation, and curettage	The lower uterine wall	Surgical repair	No complications
10	Park *et al*. 2005 [24]	36	10	***	Abdominal pain	Without	The upper portion of the left fundus	Surgical repair	N/A
11	Dabulis *et al*. 2007 [25]	N/A	9	N/A	Abdominal pain, bloating, vomiting, and diarrhea	Three previous cesarean sections	At the site of the prior cesarean section	Hysterectomy	N/A
12	TANYI *et al*. 2008 [26]	32	7	6/3	Abdominal pain, vaginal spotting	Right salpingo-oophorectomy Cesarean section curettage, Placenta percreta	3 cm from the dome of the uterus	Hysterectomy	N/A
13	Ijaz *et al*. 2011 [27]	32	8	3/1	Acute abdomen	Manual removal of placenta in a previous pregnancy	The fundus	Surgical repair	No complications
14	Visariya *et al*. 2011 [28]	26	9	2/1	Abdominal pain, giddiness, and shock	Previous cesarean	Lower anterior uterine wall	Surgical repair	No complications
15	Snigh *et al*. 2012 [29]	24	10	Primigravida	Abdominal pain	Bicornuate uterus	Left rudimentary horn	Surgical repair	No complications
16	Tola *et al*. 2014 [30]	24	8	Multigravida	Vaginal bleeding and generalized abdominal pain	Bicornuate uterus	Left part of the uterus	Left part of the uterus	No complication
17	Sinha *et al*. 2014 [31]	30	11	6/5	Syncopal attack, syncopal attack	One cesarian section, history of repair uterine rupture?	Upto the fundus	Surgical repair	No complication
18	Bandarian *et al*. 2015 [32]	30	11	4/2/ab1	Shock and acute syncopal attack	D&C, two scars	On the previous scar	Surgical repair	No complication
19	AKBAŞ *et al*. 2015 [33]	36	12	8/7	Abdominal pain	Without	At the side of the left uterine vessels extended to the cervix.	Hysterectomy	No complication
20	Taskin *et al*. 2015 [34]	32	13	2/1	Pelvic pain	Curettage, one cesarean	Posterior	Surgical repair	No complication
21	Okada *et al*. 2015 [35]	39	10	Primigravida	Abdominal pain and shock	Laparoscopic myomectomy	The fundus	Surgical repair	No complications
22	Pendy *et al*. 2015 [36]	30	8	3/2	Acute abdominal pain with hemorrhagic shock	A unicornuate uterus	Rudimentary horn	Surgical repair	No complications
23	Hefny *et al*. 2015 [37]	24	9	Primigravida	Vomiting and severe epigastric pain	Bicornuate uterus	Superior-lateral region	Surgical repair	No complications
24	Bechem *et al*. 2016 [38]	24	11	2/1	Exteriorisation of bowel through the vagina	Manual vacuum aspiration	Fundus	Surgical repair	No complications
25	Vaezi *et al*. 2017 [39]	34	12	2/1	Acute abdominal pain	Without	The fundus	Surgical repair	No complications
26	Miranda et L.2017 [40]	32	13	3/2	Acute abdominal pain	Short pregnancy interval and previous scar	The previous scar	Surgical repair	No complications
27	Surve *et al*. 2017 [41]	25	10	3/1/ab1	Shock and acute abdominal pain	One previous scar	The previous scar	Surgical repair	No complications
28	Mosad *et al*. 2017 [42]	19	11	2/1 + 0	Sudden abdominal pain, severe vaginal bleeding, syncopal attacks	IUCD	N/A	Surgical repair	No complications
29	Cho *et al*. 2017 [43]	34	7	Primigravida	Abdominal pain	Placenta percreta	Fundus	Surgical repair	No complications
30	Abbas *et al*. 2018 [44]	24	10	3/2	Shock and acute abdominal pain	Two previous scars	Posterior wall and fundus	Surgical repair	No complications
31	Abbas *et al*. 2018 [44]	27	10	3/2	Severe lower abdominal pain and shock	Two previous scars	Previous scar	Surgical repair	No complications.
32	Ambrogi *et al*. 2018 [45]	36	9	2/1	Moderete abdominal pain	One previous scar	The back and the right uterine horn	hysterectomy	No complications
33	Takashima *et al*. 2018 [46]	43	11	2/1	Sudden lower abdominal pain	Abdominal myomectomy and Cesarean section	The uterine fundus and cornea of the Cesarean scar	Hysterectomy	No complications
34	Amro *et al*. 2019 [47]	27	12	4/2 + 1	Severe lower abdominal pain	Right salpingectomy for tubal pregnancy?	The fundus	Surgical repair	No complications
35	Amro *et al*. 2019 [47]	34	6	4/1 + 3	Severe lower abdominal pain	Left-sided salpingectomy for tubal pregnancy?	The fundus	Surgical repair	No complications
36	Cecchini *et al*. 2020 [48]	35	11	5/2	Abdominal pain, fainting, and shock	Two previous cesarean sections	Lower anterior uterine wall	Surgical repair	No complications
37	Lee *et al*. 2020 [49]	28	13	2/1	Pain in the right lower quadrant, nausea, and fever	Placenta percreta	Uterine fundus	Total laparoscopic hysterectomy	No complications
38	Bruand *et al*. 2020 [50]	18	12	2/0	Abdominal pain	Abortion by vacuum extraction	Uterine horn in the right side	Surgical repair	No complications
39.	Mutiso *et al*. 2024 [3]	39	11	6/4 + 1	Severe lower abdominal pain, dizziness	Four previous hysterotomy scars	Anterior uterine rupture	Surgical repair	No complications
40	Faraj *et al*. 2022 [1]	28	9	2/1	Abdominal pain and metrorrhagia	Previous cesarean scar	In the anterior uterine wall	Surgical repair	No complications

In most cases, the rupture was localized in the fundus of the uterus (15/40).

In all cases, surgery was resorted to, whether through repair or radical excision, and in all cases, no complications were observed except for one patient who died due to hemorrhagic shock [22].

## Conclusion

Women who are pregnant and primarily in the first trimester with a history of cesarean sections should receive extra care. Uterine ruptures should be taken into mind, particularly when risk factors including a history of cesarean delivery, myomectomy, or uterine scarring are present. In addition, when symptoms like pain in the abdomen appear, uterine rupture should be considered, and it is crucial to confirm that there is no free fluid in the Douglas pouch to protect the patient.

## Supplementary Material

Video_1_rjae422

Video_legends_rjae422
