# Supplementary material for: A cesarean section scar dehiscence during the first trimester of an intrauterine pregnancy: a rare case report and literature review
Source: J Surg Case Rep. 2024 Jun 21;2024(6):rjae422. doi: 10.1093/jscr/rjae422 (PMC11190852; doi:10.1093/jscr/rjae422)
Supplement: Video_legends_rjae422 [file video_legends_rjae422.docx]

**Video legends:**

Video 1: demonstrating the surgical procedure during which the gestational sac and Placenta were removed under general anesthesia via a Pfannenstiel skin incision. During the Procedure, the patient lost an estimated quantity of 1.5 liters of blood, and numerous clots were extracted from the surgical area. Notice that some intestinal loops protruded while vacuuming the area from blood before it was restored to its normal position properly.
